# Infections and Pregnancy: Effects on Maternal and Child Health

**DOI:** 10.3389/fcimb.2022.873253

**Published:** 2022-06-08

**Authors:** Manoj Kumar, Marwa Saadaoui, Souhaila Al Khodor

**Affiliations:** Research Department, Sidra Medicine, Doha, Qatar

**Keywords:** preterm labor, miscarriage, TORCH, pregnancy complications, microbiome

## Abstract

Pregnancy causes physiological and immunological adaptations that allow the mother and fetus to communicate with precision in order to promote a healthy pregnancy. At the same time, these adaptations may make pregnant women more susceptible to infections, resulting in a variety of pregnancy complications; those pathogens may also be vertically transmitted to the fetus, resulting in adverse pregnancy outcomes. Even though the placenta has developed a robust microbial defense to restrict vertical microbial transmission, certain microbial pathogens have evolved mechanisms to avoid the placental barrier and cause congenital diseases. Recent mechanistic studies have begun to uncover the striking role of the maternal microbiota in pregnancy outcomes. In this review, we discuss how microbial pathogens overcome the placental barrier to cause congenital diseases. A better understanding of the placental control of fetal infection should provide new insights into future translational research.

## 1 Introduction

Pregnancy is a critical “formative period” that has a significant impact on an individual’s health trajectory from fetal life to adulthood (Lash, 2015). Pregnancy is governed by a series of interconnected physiological and cellular mechanisms that promote maternal homeostasis and maintain optimal maternal-fetal interface while boosting fetal growth (Ander et al., 2019). These mechanisms enable the woman’s body to undergo, physiological and immunologic adaptations to host fetal antigens. From the mother’s immune system perspective, the fetus is an allograft that contains foreign antigens from the father (Robinson and Klein, 2012). To protect the fetus from immune rejection, the maternal immune must strike a delicate balance between maintaining tolerance to the fetal allograft by inducing anti-inflammatory properties at the maternal-fetal interface and maintaining an elevated inflammatory response with rising levels of pro-inflammatory cytokines at mucosal surfaces such as the gut to protect against microbial challenges (Koren et al., 2012; Erlebacher, 2013; PrabhuDas et al., 2015; Nuriel-Ohayon et al., 2016; Marchant et al., 2017). Concurrently, the transition of the maternal immune system during pregnancy from more inflammatory states at the start of pregnancy to lower levels of inflammation in mid-pregnancy makes pregnant women more vulnerable to infections (Mor and Cardenas, 2010) and pregnancy complications. Although the exact etiology of pregnancy complications remains elusive, the complex interaction of microbial or other factors with host immune system is thought to be the underlying pathogenesis of pregnancy complications (Megli and Coyne, 2021).

The emerging findings from the various pregnancy cohorts (Piler et al., 2017; Pansieri et al., 2020), as well as many animal studies, demonstrated that pregnancy complications are heterogeneous and depend on a variety of factors, including intra- or extra-uterine infection, microbial dysbiosis, and aberrant immune system (Romero et al., 2014a; MacIntyre et al., 2015; Waken et al., 2017; Fettweis et al., 2019; Serrano et al., 2019; Jehan et al., 2020; Kumar et al., 2021a). During pregnancy, multiple immune signaling pathways and cytokines normally act as mediators to promote a healthy and successful pregnancy and to arbitrate defense against pathogens (Mor and Cardenas, 2010). However, the complexity of interaction between multiple host factors, including maternal infection or aberrant activation of the immune response during pregnancy, could lead to severe pregnancy complications and have a negative impact on pregnancy health or the developing fetus (Kumar et al., 2021a). Indeed, the emerging evidence indicates that these pregnancy complications may pose significant challenges to fetal growth and development during pregnancy, as well as susceptibility to a variety of diseases later in life (Rahman et al., 2012; Nimeri et al., 2013).

In this article, we review the complexity of the interaction between various host factors associated with different maternal infections and dynamic fluctuation of the maternal immune system in both inducing pregnancy complications and eliciting detrimental effects on the developing fetus.

## 2 Maternal Infections During Pregnancy

Complications from various bacterial, viral, parasitic or fungal maternal infections can occur at any stage of pregnancy. Indeed, several studies suggest that pregnant women are more vulnerable to certain infections as a result of compensatory physiological and immunologic adaptations. The “TORCH” pathogens including *Toxoplasma gondii*, Other agents (syphilis, varicella-zoster, parvovirus B19), Rubella, Cytomegalovirus (CMV), and Herpes simplex virus, are known to cause various pregnancy complications such as congenital infections, abortion, and intrauterine fetal growth restrictions (Megli and Coyne, 2021). In addition to these most common infections linked to congenital defects, ZIKA infection, one of the newest TORCH pathogens, has recently sparked public concern, resulting in severe pregnancy complications ranging from fetal growth restriction to miscarriages in 2015-2017 (Coyne and Lazear, 2016). Most TORCH pathogens cause mild to moderate morbidity, but infections during pregnancy can have serious fetal consequences due to stimulation of systemic or local factors (**Table 1**). Emerging studies indicate that various microbial pathogens and neurotropic viruses can cross the placenta barrier, and an aberrant immune response to pathogens can cause various pregnancy complications (Platt et al., 2018), such as:

- Acute maternal infection during pregnancy: may cause maternal morbidity and/or mortality or a wide range of obstetric complications, including low birth weight, stillbirth, miscarriage, and preterm labor.
- Vertical transmission during pregnancy: which can result in congenital infection, intrauterine death, or permanent disability.
- Perinatal transmission during delivery: which can lead to severe neonatal diseases.

**Table 1:** Pathogens associated with pregnancy complications and their pathological role in adverse pregnancy outcomes.

Pathogen	Transmission	Maternal symptoms	Immune response associated with infection	Pregnancy complications	Reference
**Bacteria** *Listeria monocytogenes*	Consumption of contaminated food	Fever, Flu-like symptoms, headache, vomiting	IFN-γ, IL-1β, IL-10	Vertical transmission, congenital disease, Miscarriage, stillbirths, fetal death	(Teixeira and Kaufmann, 1994; Thomas et al., 2020)
*Brucella species*	Consumption of contaminated food or contact with infected animal	Fever, join and muscle pain	IL-6, IL-8, MCP-1	Spontaneous abortions, preterm birth, chorioamnionitis	(Fernandez et al., 2016; Bosilkovski et al., 2020)
*Chlamydia trachomatis*	Sexual contact with infected person	Vaginal discharge, pelvic or abdominal pain	IL-1α, IL-6, IL-8, TNF-α, IFN-γ,	Premature rupture of membrane, Preterm, fetal eye infection	(Brunham and Rey-Ladino, 2005; Adachi et al., 2016)
*Neisseria gonorrhoeae*	Sexual contact with infected person	Vaginal discharge and bleeding, Painful urination, painful bowel movements	IL-1β, IL-6, IL-8, TNFα, MCP-1	Premature rupture of membrane, Preterm birth, low birth weight	(Lenz and Dillard, 2018; Vallely et al., 2021)
*Treponema pallidum/Syphilis*	Sexual contact with infected person	Fever, Swollen lymph nodes, headache and joint pains	IL-2, IFN-γ,TNFα	Vertical transmission, still birth, pregnancy loss, low birth weight	(Wicher and Wicher, 2001; Cerqueira et al., 2017)
*Streptococci group B* *S. pneumoniae*	Commensal Contaminated air	Normally no symptoms, but some women can have low grade fever, fast or slow heart rate and breathing rate, lethargy, Urinary tract infection	IL-1β, IL-8, IL-10, TNF-α	Vertical transmission (rare), Vertical transmission during delivery, preterm birth, neonatal sepsis	(Patras and Nizet, 2018; Flaherty et al., 2019; Phillips and Walsh, 2020)
***Bacterial vaginosis*** *E. coli*	Commensal	Diarrhea, abdominal cramps, vomiting, fatigue, Urinary tract infection	IL-1β, IL-8, IL-10, TNF-α, IFN-γ	Preterm rupture of membranes, preterm birth, still birth	(Sacerdoti et al., 2018; Wilkie et al., 2019; Glaser et al., 2021; Megli and Coyne, 2022)
*Gardnerella vaginalis*	Sexual contact with infected person	Vaginal discharge, infection with fishy odor	IL-1β, IL-6, TNF-α,	Vertical transmission (no evidence), Preterm rupture of membranes, low birth weight, preterm birth	(Wong et al., 2018)
*Trichomonas vaginalis* *Ureaplasma urealyticum* *Mycoplasma hominis*	Sexual contact with infected person	Vaginal discharge, itching in the genitals	IL-1β, IL-6, IL-8	Premature rupture of membrane, Preterm birth, low birth weight	(Cauci and Culhane, 2007; Capoccia et al., 2013; Margarita et al., 2020)
**Viruses** Cytomegalovirus (cmv)	Ingestion of infected body fluids (blood, saliva, urine, breast milk, feces)	High fever, aching muscles, skin rash, sore throat	CXCL-10 (blood) TNF-α, IL-1β, IL-10, IL-12, IL-15, IL-17, CCL-2, CCL-4, CXCL-10 (amniotic fluid)	Vertical transmission, congenital disease, preterm birth, Fetal hearing loss, vision loss, intracranial calcifications	(Cannon et al., 2011; Scott et al., 2012; Liu et al., 2021)
Herpes simplex virus	Sexual or oral contact with infected person	Genital herpes, rash, cold sores on lips, gums	Anti-HHV-IgG, IgM	Vertical transmission during delivery, Spontaneous abortion, miscarriage, chorioretinitis, intracranial calcification in neonates	(Pinninti and Kimberlin, 2013; James et al., 2014)
Rubella	Contaminated respiratory droplets	Low-grade fever, headache, sore throat, conjunctivitis	Anti-rubella-IgG, IgM	Miscarriage, still birth, vertical transmission, fetal ocular disorder, auditory or speech disorder and autism	(Wilson et al., 2006; Arora et al., 2017; Yockey and Iwasaki, 2018)
HIV	Sexual or contaminated material	Weight loss, chronic diarrhea, night sweats, rash and increased susceptibility of infections	IL-1β, IL6, IL10, CD4+ ↑ IFNα ↓	Vertical transmission, congenital disease, neonatal high mortality and lifelong devastating effect, cardiovascular diseases and increased risk to infections	(Maartens et al., 2014; Johnson and Chakraborty, 2016; Moncunill et al., 2020)
Zika virus	Aedes species, sexual, blood borne	Fever, joint and muscle pain, rash	IL-6, IL-15, IL-17, IFN-γ, IFN-α, TNF-α (blood)	Pregnancy loss, still birth, congenital disease, neurological defects including intracerebral calcifications, enlarged ventricles and collapsing brain, echogenic bowel,	(Ornelas et al., 2017; Maucourant et al., 2019)
SARS-CoV2 MERS	Respiratory or contact with infected material	Fever, cough, tiredness, loss of taste or smell	IL1, IL2, IL-7, IL10, TNF-α	Vertical transmission (no evidence), maternal mortality, preeclampsia, preterm birth	(Alfaraj et al., 2019; Kumar and Al Khodor, 2020; Saadaoui et al., 2021; Villar et al., 2021)
Hepatitis C virus	Ingestion of infected material	Cholestasis, itching, yellow eye or skin	CXCL-11, CXCL-12	Vertical transmission (rare), Vertical transmission during delivery, low birth weight, preterm birth, neonatal chronic liver disease	(Chudnovets et al., 2020)
Varicella-zoster virus	Contaminated respiratory droplets	Red rash, blisters, itching	IL-1α, IL-6, CXCL10, TGF-β	Vertical transmission (rare), Vertical transmission during delivery, Limb and gastrointestinal abnormalities	(Chudnovets et al., 2020; Nanthakumar et al., 2021)
Parvovirus B19 (Fifth disease)	Contaminated respiratory droplets	Mild fever, sore throat, red rash	IL-2, IL-12, IL-15, IFN-γ	Anemia, still birth, pregnancy loss	(Isa et al., 2007; Adams Waldorf and McAdams, 2013)
Influenza	Contaminated respiratory droplets	Fever with chills, cough, sore throat, runny or stuffy nose, body aches, headache	TNF-α, IL-1β, IL-6, IL-15, IFN-γ	Low birth weight	(Le Gars et al., 2016)
Enterovirus	Ingestion of infected material	Diarrhea, conjunctivitis or rash		Increased risk of type 1 diabetes in childhood	(Adams Waldorf and McAdams, 2013)
West Nile virus	Bite of infected mosquito Arbovirus (Culex species)	Fever, vomiting, neck stiffness, or seizures	IL-2, IL-4, TNF-α, IFN-γ	Meningitis/encephalitis, possible lissencephaly	(Stewart et al., 2013; Zidovec-Lepej et al., 2021)
**Protozoa** *Taxoplasma gondii*	Ingestion of contaminated food or oocysts	Usually cause no symptoms, but some infected people show symptoms, such as, Fever, aching muscles, tiredness, sore throat	IFN-γ, IL-12, IL-17 (blood) IL-4, IL-10, TGF-β (placenta)	Miscarriage, stillbirth, vertical transmission, congenital toxoplasmosis (blindness, deafness, intracranial calcifications)	(Abou-Bacar et al., 2004; Zhang et al., 2015)
*Plasmodium falciparum* *Plasmodium vivax*	Arthropod vector (*Anopletes* species)	Fever, shaking chills, headache, muscle aches, vomiting, diarrhea	IFN-γ, TNF-α, IL-10	Severe hypoglycemia, Fetus growth restriction, low birth weight, miscarriage, preterm, vertical transmission (rare)	(Artavanis-Tsakonas et al., 2003; Nasr et al., 2014; Romero et al., 2021; Chua et al., 2021) (Nasr et al., 2014; Briand et al., 2016; Cutts et al., 2020; Romero et al., 2021; Lee et al., 2021)
**Fungi** *Candida albicans* *Candida parapsilosis*	Normal vaginal flora, but during pregnancy *Candida* can cause infection due to microbial dysbiosis or vaginal hormonal fluctuation	Itching, burning, thick, white vaginal discharge	IL1β, IL8	Low birth weight, fetal candidiasis, premature rapture of membrane	(Maki et al., 2017; Ardizzoni et al., 2021)

Bacteria; Virus; Protozoa; Fungi.

To better understand the pathophysiology and consequences of TORCH pathogens and other maternal infections during pregnancy, as well as their impact on pregnancy outcomes, we classified these pathogens into the following categories:

### 2.1 Bacterial Infections

Acute bacterial infections during pregnancy can increase pregnancy complications and even have a negative pregnancy outcome (**Table 1**). Bacterial infections, such as listeriosis, bacterial vaginosis, and sexually transmitted infections (STIs), can be caused by a single bacterial pathogen or by a microbial dysbiosis and can result in inflammasome signaling at the maternal-fetal interface and/or severe congenital anomalies in the developing fetus.

#### 2.1.1 Listeriosis

Listeriosis is a foodborne bacterial infection caused by *Listeria monocytogenes* (Wang et al., 2021). Although this infection is uncommon in healthy people, pregnant women are particularly vulnerable to *L. monocytogenes* infection, possibly due to their altered immune status (Wang et al., 2021). Once transmitted through contaminated food, *L. monocytogenes* can cross the intestinal barrier to reach the placenta causing pregnancy complications such as preterm birth, stillbirth, congenital diseases, and sepsis (Mateus et al., 2013). A recent listeriosis outbreak in South Africa reported exceptionally high mortality rates among infected infants (>28%) and pregnant women (Thomas et al., 2020). Although the pathophysiology of *L. monocytogenes* placental transmission is still largely unknown, emerging studies show that the bacterium binds to E-cadherin on primary trophoblasts *via* the internalis protein InIA and InIB or InIP (Disson et al., 2008; Faralla et al., 2018), to survive in a hostile environment, suggesting that the bacterium uses trophoblast-specific virulence factors for placental colonization and fetal tissues infection (Bakardjiev et al., 2006). Concurrently, bacterial colonization in placental tissues leads to abscess development, innate immune cells recruitment, and aberrant IFN-γ secretion at the maternal-fetal interface (Charlier et al., 2020; Maudet et al., 2021) and subsequently stimulates inflammasome signaling and increases severity of neonatal outcomes. **A.**

#### 2.1.2 Bacterial Vaginosis

Bacterial vaginosis (BV) is characterized by the loss of healthy vaginal microbiome composition and an increase in the abundance of pathogenic microbes (Isik et al., 2016). BV is the most common gynecological infection among women during reproductive age and pregnancy (Isik et al., 2016; Kumar et al., 2021a), resulting in serious pregnancy complications such as miscarriage and preterm birth (**Table 1**) (Leitich et al., 2003). Vaginal infections caused by group B *Streptococcus* (GBS), *Escherichia coli*, *Bacteroides* species, *C. trachomatis*, and *N. gonorrhoeae* can ascend to the genital tract and intraamniotic fluid causing chorioamnionitis (Galinsky et al., 2013; Jain et al., 2022). Infections caused by ascending genito-urinary tract pathogens are typically polymicrobial (Mendz et al., 2013) and often associated with microbial biofilm and antimicrobial cervical mucous plug to reach the intra-amniotic fluid or maternal-fetal interface and induce inflammation locally, which then endangers the fetus due to aberrant inflammation at the fetal membrane (Ayala et al., 2019). There is no clear evidence of how dysbiotic flora crosses the maternal barriers to reach the fetus, but *GBS* and *E. coli* are the most common pathogens found in the placenta and late-onset sepsis in neonates (Wilkie et al., 2019; Glaser et al., 2021). GBS and *E. coli* can both adhere to the fetal membrane *via* various virulence factors and stimulate neutrophils and macrophages to produce inflammatory cytokines and potentially develop extracellular traps to cause premature fetal membrane rupture (Armistead et al., 2020; Coleman et al., 2021; Deshayes de Cambronne et al., 2021).

#### 2.1.3 Sexually Transmitted Infections

Changing the vaginal microenvironment during pregnancy may increase vaginal susceptibility to opportunistic STIs, which are frequently asymptomatic, but can cause severe pregnancy complications if left untreated. Ascending transmission of *Chlamydia trachomatis* and *Neisseria gonorrhoeae* can lead to pelvic inflammatory disease and endocarditis, as well as serious pregnancy complications like ectopic pregnancy, preterm birth, and low birth weight (Adachi et al., 2016; Heumann et al., 2017). Syphilis is another common STI (caused by *Treponema pallidum*). Although the pathophysiology of *T. pallidum* ascending transmission is unknown, it may be dependent on both the gestational age of the fetus and the maternal stage of infection (Kimball et al., 2020; Primus et al., 2020). Vertical transmission of this bacterium can cause excessive inflammation at the maternal-fetal interface resulting in mild to severe pregnancy complications such as low birth weight, preterm birth, congenital anomalies, and sometimes fetal loss (Primus et al., 2020; Megli and Coyne, 2021).

#### 2.1.4 Maternal Microbiome

**Figure 1:**
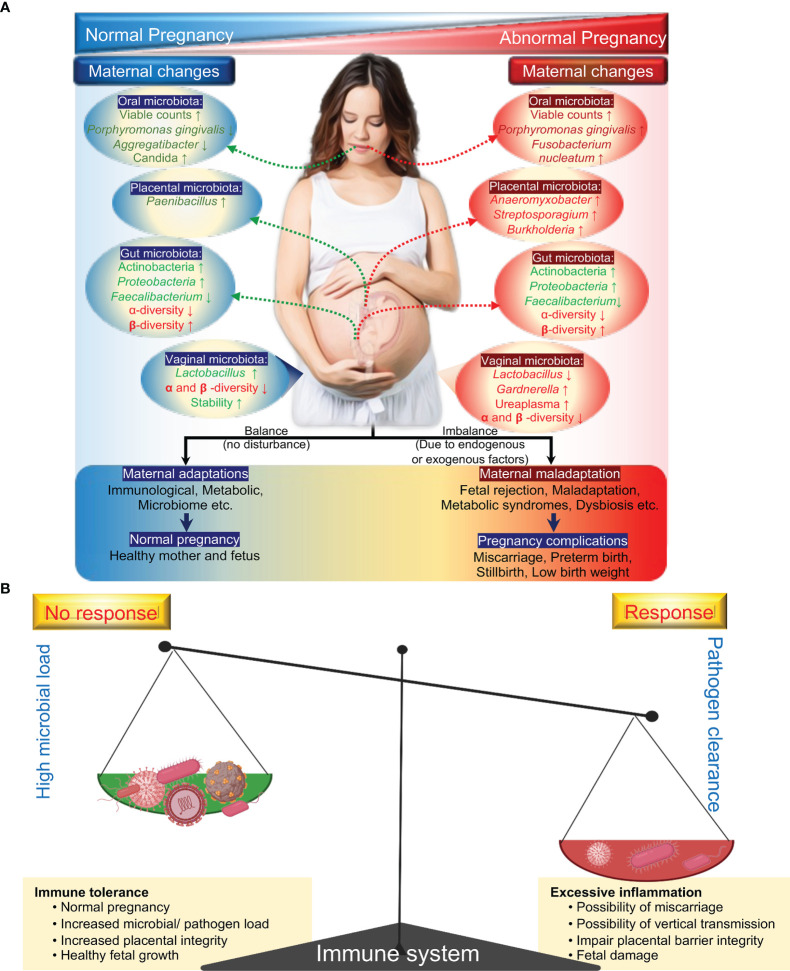
General microbial dynamics during health pregnancy and complicated pregnancy. Known changes in the microbial composition: changes in a specific taxonomy (green) and changes in community diversity (red) **(A)**. Immune response during pregnancy: double-edged sword **(B)**. During pregnancy, the maternal immune system has to balance between sustaining the growth of the fetus and protecting both mother and fetus from pathogens.

The maternal microbiome undergoes significant changes during the course of pregnancy and has been suggested to play an influencing role in the health of pregnant women and their neonates during pregnancy and beyond (Prince et al., 2015; Fettweis et al., 2019). The maternal microbiome consists of distinct microbial communities dominated by different bacterial taxa. For example, a vaginal microbial community dominated with *Lactobacillus* species are suggested to be associated with a healthy pregnancy, whereas the abundance of a complex vaginal microbial community of CST-IV including *Gardnerella*, *Prevotella, Chlamydia* and bacterial vaginosis (BV)-associated bacterium-I (BVAB-I) are associated with increased risk for adverse pregnancy outcomes and fetal infection (Ravel et al., 2011; Kumar et al., 2021a; Saadaoui et al., 2021). The gut and oral microbial communities, like the vaginal microbiome, undergo significant changes during pregnancy, including a significant decrease in alpha diversity and a significant enrichment in Actinobacteria and Proteobacteria species in the gut and oral environment (**Figure 1A**) (Offenbacher et al., 2006; Aagaard et al., 2012).

To ensure healthy pregnancy outcomes, this delicate balance between microbial communities and immune tolerance or immune response must be maintained (**Figure 1B**). Numerous studies have suggested that microbial dysbiosis is linked to a variety of pregnancy complications and fetal development (Seong et al., 2008; Han et al., 2010). For example, abnormal changes in the oral microbiota during pregnancy, such as a decrease in *Lactobacillus species* or an increase in the abundance of *Porphyromonas gingivalis*, may lead to further infections and the production of pro-inflammatory cytokines, which is thought to be a contributory factor to various pregnancy complications such as early labor, pregnancy loss, and low birth weight, among others (Aagaard et al., 2012; Koren et al., 2012; de Weerth et al., 2013; Romero et al., 2014b; DiGiulio et al., 2015; Goltsman et al., 2018). While the link between microbial dysbiosis and pregnancy complications is clear, the exact nature of these interactions is unknown. It is unclear whether dysbiosis impairs the maternal immune system or influences other mechanisms (Zhang et al., 2015; Kumar et al., 2020) to promote pregnancy complications and fetal development. These findings suggest that intra- or extra-uterine infection or vaginal dysbiosis induces an abnormal immune response in pregnant women and may be an important predictor marker for adverse outcomes of congenital infections.

### 2.2 Viral Infections

The human microbiome has a significant virome component, which includes a diverse collection of endogenous retroviruses, eukaryotic viruses, and bacteriophages (Wylie et al., 2012), and is increasingly recognized as an orchestrator of bacterial diversity and functionality (Mills et al., 2013; Barr, 2017). Although the majority of viruses are harmless, some pathogenic viruses can cross the maternal-fetal interface and influence placental functions, potentially causing fetal disease (**Table 1**).

#### 2.2.1 Cytomegalovirus

Cytomegalovirus (CMV) is a DNA virus that belongs to the Herpesviridae family. CMV is the most common viral infection transmitted vertically *in utero*, causing a wide range of congenital disorders such as hearing and vision loss, intracranial calcifications, microcephaly, organ dysfunction, and intellectual disability (Liu et al., 2021). CMV is typically transmitted from person to person *via* infected bodily fluids such as blood, saliva, urine, and breast milk (Cannon et al., 2011). Once infected, the virus can live in bone marrow hematopoietic cells for the rest of one’s life (Collins-McMillen et al., 2018). However, it is a primary infection during pregnancy, rather than a reactivation of a persistent infection, that causes adverse pregnancy outcomes (Boppana et al., 2001; Maidji et al., 2006). Although the exact pathophysiology of CMV is unknown, the severity of the infection and fetal consequences are dependent on gestational age at the time of maternal infection, implying that changes in maternal immune status and the maternal-fetal interface play an important role in CMV vertical transmission. According to new research, CMV may first infect placental pericytes before infecting the fetus (Aronoff et al., 2017). Additionally, CMV infected pregnant women have elevated level of cytokines including TNF-α, IL-1β, IL-10, IL-12, IL-15, IL-17, and CXCL10 which may cause various pregnancy complications or serious health problems to the baby, such as preterm birth or low birth weight, or hearing loss at birth or later in life, depending on the pregnancy (Scott et al., 2012).

#### 2.2.2 Herpes Simplex Virus

Herpes simplex virus (HSV) infections are often asymptomatic or cause mild symptoms in adults; however, the changing maternal immune system from higher inflammatory status at the beginning of pregnancy to a lower level of inflammation in mid-pregnancy may predispose the pregnant women to different viral infections, including HSVs (Straface et al., 2012). Although the mechanism of its transplacental transmission is unknown, vertical transmission *via* direct contact with viral lesions in the genital tract during delivery is a more common route of neonatal infection (James et al., 2014). As a result, maternal HSV infection near the time of delivery increases the risk of vertical transmission, which can result in herpes simplex encephalitis, chorioretinitis, and intracranial calcification in neonates, with a 50-80% mortality rate in untreated cases (Pinninti and Kimberlin, 2013).

#### 2.2.3 Rubella Virus

Rubella virus is a contagious virus in the Togaviridae family. Rubella virus is primarily transmitted *via* respiratory droplets, and in healthy adults, the infection causes mild illness with a low-grade fever; however, pregnant women who acquire rubella infection are 85 percent more likely to have a miscarriage or stillbirth, and the virus can induce necrosis in the syncytiotrophoblasts allowing it to cross the placental barrier (Lambert et al., 2015; Arora et al., 2017). The neonatal infection can cause severe birth defects with devastating, lifelong consequences such as ocular disorder, auditory problems, cardiovascular defects, speech disorder, and autism (Lambert et al., 2015).

#### 2.2.4 Human Immunodeficiency Virus

Despite the availability of effective anti-HIV therapies, approximately 38 million people are still infected with HIV; among these 53% are women (Data, 2020). HIV can be transmitted through the placenta, perinatally (from direct contact to maternal vaginal fluids or blood during delivery), or postnatally (from breast milk or other sources) (Milligan and Overbaugh, 2014). As a result, congenital HIV transmission remains the leading cause of neonatal infections and the associated neonatal mortality or life-long devastation. Although it is unknown how HIV crosses the placental barrier, neonates born to HIV-infected women are always at a significantly high risk of vertical transmission (25 percent in the absence of antiretroviral therapy) (Bernstein and Wegman, 2018), which predispose them to serious health consequences, including developing acquired immunodeficiency syndrome (AIDS) and cardiovascular diseases (Maartens et al., 2014). Additionally, HIV infection is often associated with opportunistic infections, further increasing the risk of adverse pregnancy outcomes or vertical transmission (Johnson and Chakraborty, 2016).

#### 2.2.5 Zika Virus

Zika virus (ZIKV) is an emerging arbovirus that is endemic in Africa, America, Asia, and Europe (Khaiboullina et al., 2018). ZIKV is primarily transmitted by the bite of an infected mosquito (Khaiboullina et al., 2018). Though ZIKV infection in adults causes mild symptoms with low-grade fever, headache, rash (Javanian et al., 2018), infection during pregnancy can cross the placenta and increase the risk of adverse pregnancy outcomes and postnatal developmental sequelae, such as miscarriage or stillbirth, or surviving infants show lifelong neurological defects such as enlarged ventricles, collapsing brains, and microcephaly. Emerging studies indicate that ZIKV can selectively infect decidual fibroblasts and macrophages, trophoblasts, hofbauer cells (fetal macrophages), and umbilical cord (Quicke et al., 2016; Tabata et al., 2016) and can significantly induce cytokine levels of IL-6, IL-15, IL-17, IFN-α, CXCL10 and IFN-γ at the maternal-fetal interface and in amniotic fluid, which may result in severe fetal neurological abnormalities (Ornelas et al., 2017; Maucourant et al., 2019). Accumulating evidence shows a link between ZIKV infection and congenital microcephaly (Tang et al., 2016; Gladwyn-Ng et al., 2018). ZIKV infection during gestation can trigger endoplasmic reticulum stress in the embryonic brain, which may perturb physiological unfolded protein response in the cerebral cortex and lead to microcephaly in the babies born from mothers infected with ZIKV (Mlakar et al., 2016; Gladwyn-Ng et al., 2018).

#### 2.2.6 COVID-19

The most recent COVID-19 pandemic, caused by the Severe Acute Respiratory Syndrome Coronavirus 2 (SARS-CoV-2), infected over 308 million subjects, and killed 5.5 million people worldwide, highlighting the importance of focusing on women’s health. SARS-CoV2 is primarily spread through close contact with an infected person, as well as through aerosols and respiratory droplets (Saadaoui et al., 2021) and can severely impact a variety of physiological and immunological processes, including pregnancy health and outcomes (Kumar and Al Khodor, 2020; Saadaoui et al., 2021). SARS-CoV-2 binds to host cells through the angiotensin-converting enzyme 2 (ACE2) receptor (Yan et al., 2020), which is expressed on the surface of various trophoblasts including, cytotrophoblast and syncytiotrophoblast cells at the maternal-fetal interface (Gengler et al., 2021). Although the virion genome has been observed in placental and vaginal samples (Dong et al., 2020), but the majority of recent reports show no evidence of vertical transmission (Saadaoui et al., 2021), suggesting that SARS-CoV2 cannot cross the placental barriers even in severely infected women. Despite the magnitude of the pandemic, pregnant women do not appear to vertically transfer the SARS-CoV2 to the fetus, but the inflammatory storm during SARS-CoV2 infection might indirectly induce pregnancy complications and even fetal developmental obstacles. For example, increasing levels of inflammatory cytokines during infection, such as IL-1, IL-2, IL-7, IL-10, and TNF-α in the maternal blood, at the maternal-fetal interface may lead to adverse pregnancy complications, including maternal mortality, preeclampsia, and preterm birth (Villar et al., 2021).

### 2.3 Parasites

Despite the fact that emerging knowledge and practices on prevention of mosquito-borne diseases have significantly reduced parasitic infections worldwide (Nguyen-Tien et al., 2021), some parasitic infections are still common during pregnancy due to the living conditions (Brummaier et al., 2019) or decreased host immunity. Due to reduced maternal immunity during pregnancy, parasitic infections are common among pregnant women living in low resource settings (Brummaier et al., 2019) and therefore can influence maternal and fetal health (**Table 1**).

#### 2.3.1 Toxoplasmosis

Toxoplasmosis is caused by *Toxoplasma gondii* resulting in more than 200,000 cases of congenital toxoplasmosis worldwide each year (Bigna et al., 2020). *T. gondii* can be vertically transmitted during pregnancy to cause toxoplasmosis and can lead to a high risk of congenital diseases (Bigna et al., 2020). Although, vertical transmission of toxoplasmosis can occur only in 30-40% of patients, but *T. gondii* infection during pregnancy could lead to an aberrant immune response in blood to control the infection (Sasai and Yamamoto, 2019). Immune response toward the *T. gondii* infected cells leads to aberrant production of IFN-γ, IL-12, IL-17 which can result in miscarriage and stillbirth (Smith et al., 2021).

#### 2.3.2 Malaria

Malaria parasites, mainly *Plasmodium falciparum* and *Plasmodium vivax*, are other pathogens associated with an elevated risk of pregnancy complications, including fetal growth restriction and preterm birth (Briand et al., 2016; Romero et al., 2021). Malaria parasite-infected erythrocytes during pregnancy can adhere to placental receptors and trigger placental inflammation and subsequent damage, causing harm to both mother and her infant (Chua et al., 2021). Emerging evidence suggests that malaria parasite-infected women have significantly higher systemic levels of pro-inflammatory cytokines and chemokines, including TNF-α, IFN-γ, IL-10, which appear to be a key mediators of pregnancy complications (Nasr et al., 2014). IFN-γ response during pregnancy is a double-edged sword. It plays both protective and pathological roles during malaria infection (Nasr et al., 2014). IFN-γ response in malaria parasite-infected women is crucial for parasite clearance in both the liver and blood stages (Inoue et al., 2013), however high levels of IFN-γ may also exacerbate the disease severity, including cerebral malaria and other pregnancy complications such as embryotoxicity or abnormal placenta as shown in **Figure 3** (King and Lamb, 2015).

### 2.4 Fungal Infections

The vast majority of fungi are harmless, and serious fungal infections are uncommon during pregnancy; however, they may occur with higher frequency in pregnant women, which potentially can increase maternal complications, including prematurity or, in some cases, even fetal loss (Rasti et al., 2014).

#### 2.4.1 Candidiasis

Candidiasis is the most common cause of infection worldwide and is caused by Candida, an opportunistic yeast (Manolakaki et al., 2010). Under normal conditions, most *Candida species* are commensals or endosymbionts, but some species, such as *Candida albicans* and *Candida parapsilosis*, can cause candidiasis (AN and Rafiq, 2021). Vaginal candidiasis is the most common gynecological infection during reproductive age and pregnancy. According to emerging studies, up to 40% of women have vaginal colonization with *Candida* spp. during pregnancy (DiGiulio, 2012), which can easily transmit to the maternal-fetal barrier and progress to intra-amniotic infection which may lead to severe pregnancy complications including low birth weight or fetal candidiasis (Siriratsivawong et al., 2014; Drummond and Lionakis, 2018).

## 3 Pregnancy Complications Associated With Maternal Infections

**Figure 2:**
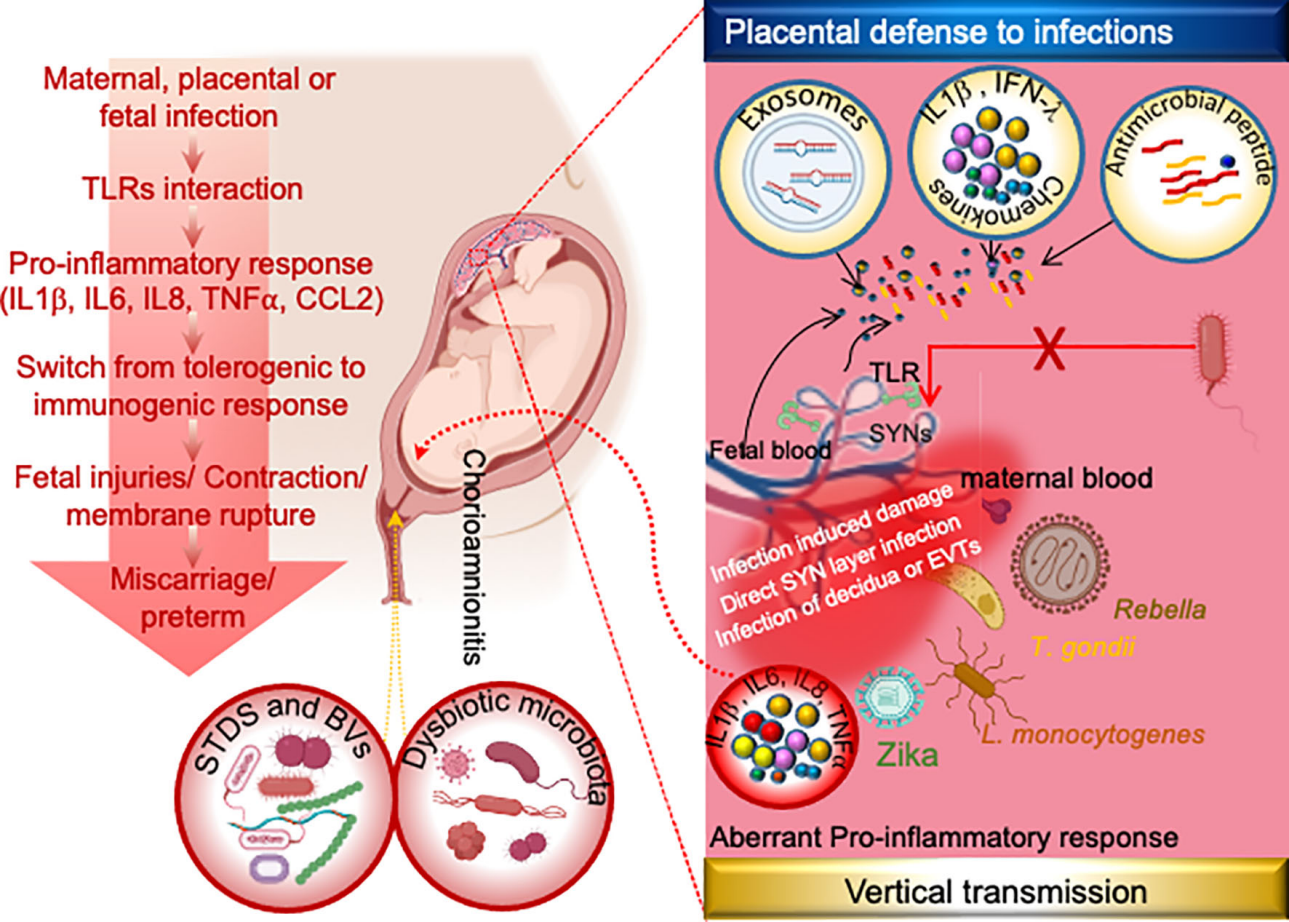
Mechanism of placental physical and immune defense and possible mechanism of vertical transmission of TORCH and other pathogens during pregnancy. The human placenta has evolved several layers of defense including antimicrobial effectors such as exosomes, antimicrobial peptides, and/or innate immune response to infection by release of cytokines and/or highly integrated syncytiotrophoblast. Syncytiotrophoblast is the placental barrier between maternal and fetal blood that allows selective exchanges in nutrients and gases between the embryo and the mother but inhibits the microbial invasion. Although the exact mechanisms by which TORCH pathogens cross the placental barrier are still unclear. However emerging studies indicates these pathogens reach the fetus through infected maternal decidua, infected extracellular trophoblasts (EVTs) and/or through direct infection of the syncytium, while the vaginal pathogens gain access to the amniotic cavity *via* ascending transmission. Following the amniotic cavity infection, toll-like receptors (TLRs) at the fetal-maternal interface get activated and induce pro-inflammatory cytokines and chemokines, leading to further immune cells recruitment. Switching of maternal immune response from tolerogenic to inflammatory state leads to the premature activation of cervical ripening proteins and onset of labor. *Common STDs and BVs infections are C. trachomatis, N. gonorrhoeae, T. pallidum, GBS, E. coli etc. Dysbiotic microbiota infections are GBS, E. coli, C. trachomatis, Gardnerella, Prevotella etc. EVTs: Extravillous trophoblasts, GBS: Group B Streptococcus, SYNs:* syncytiotrophoblast.

Although complications caused by maternal infections or extrinsic abnormalities can occur at any stage of pregnancy, the first trimester is critical for placental development and the formation of a selective barrier between maternal and fetal tissue (Burton et al., 2016). The placental barrier, which is made up of multiple layers of maternal and fetal tissues, serves as a strong barrier against human pathogens reaching the fetus (Burton et al., 2016). Syncytiotrophoblasts (SYNs) are multinucleated cells that form a strong barrier between maternal and fetal blood within the placenta (Ander et al., 2019). Despite the fact that SYNs are highly resistant to bacterial or viral infections and produce type III IFNs (Ander et al., 2019), some pathogens can still cross these barriers and reach the fetus (**Figure 2**). Although the mechanism(s) by which pathogens breach the strong barriers remains unknown, intrauterine infection and associated inflammation are significant contributors to pregnancy complications. Surprisingly, approximately 25% of preterm births are microbially induced, either through intrauterine infection or maternal extrauterine infection (Agrawal and Hirsch, 2012).

## 4 Pregnancy Complication as a Result of Aberrant Immune Response

**Figure 3:**
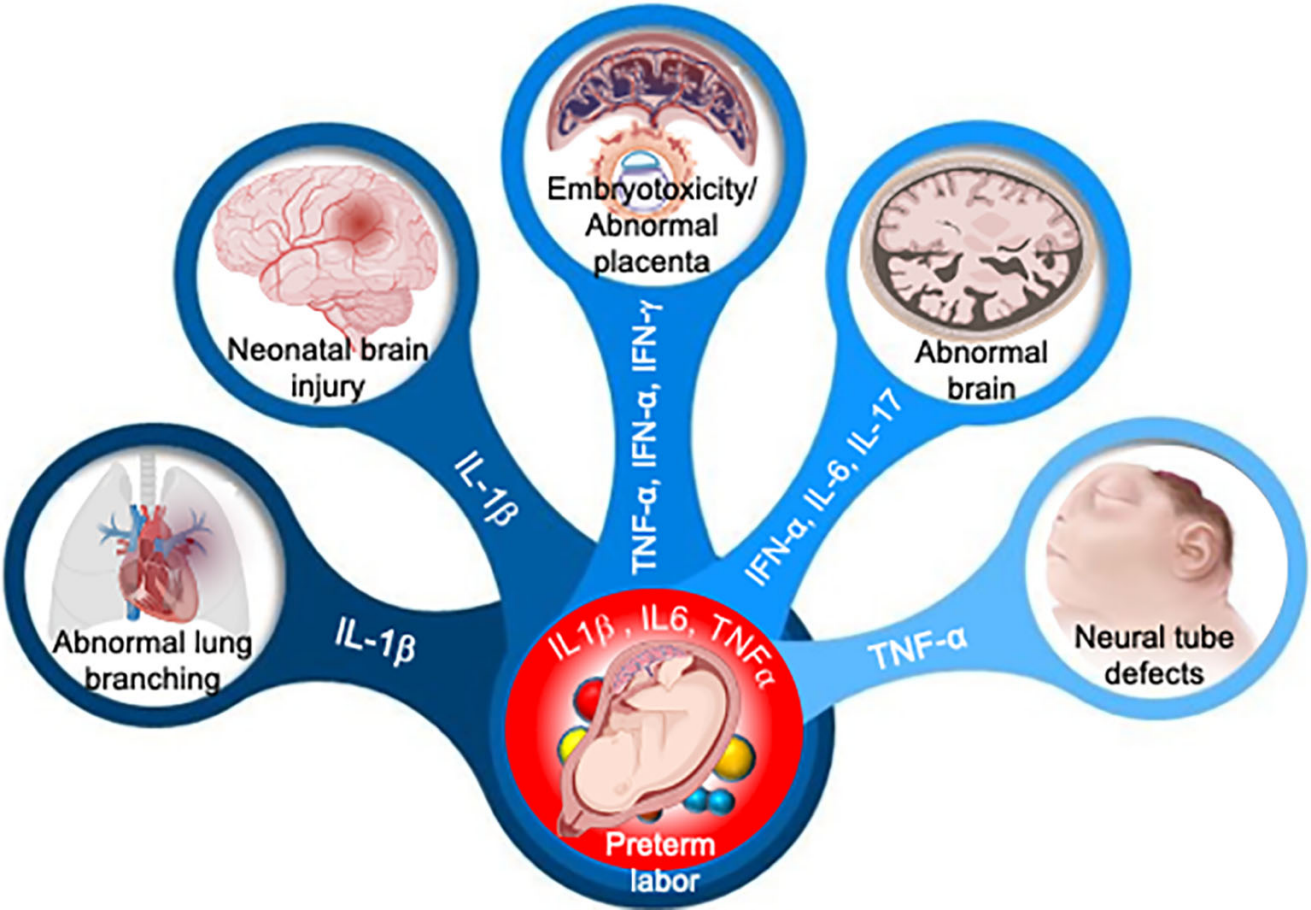
Adverse pregnancy outcomes induced by aberrant cytokine response. Aberrant levels of IL-1β, IL-6, IL-17, TNF-α, IFN-α and IFN-γ in amniotic fluid can induce multiple organ development failure in fetus or induce premature activation of cervical ripening proteins and onset of preterm labor.

According to the findings of recent pathological and advanced metagenomic studies, which have been supplemented by cellular and experimental animal studies, a significant amount of pathogens can bypass the placental barrier integrity and modulate an abnormal immune response at the maternal-fetal interface or in the amniotic fluid (Megli and Coyne, 2021). Microbial pathogens commonly associated with periodontal disease or found in the lower genital tract can cross the placental barrier and react to amniotic fluid in women who had preterm labor, possibly *via* hematogenous dissemination *via* the transplacental passage or ascending microbial invasion into the amniotic fluid (chorioamnionitis) from the urinary tract (Cobb et al., 2017). Normally, microbial-induced pregnancy complications are mediated by an aberrant inflammatory process. Many studies have revealed an elevated level of proinflammatory cytokines such as IL-1, IL-6, IL-8, and TNF-α in cervicovaginal lavage or amniotic fluid of women experiencing pregnancy complications (Agrawal and Hirsch, 2012; Romero et al., 2014a; Gee et al., 2021). Interestingly, emerging evidence suggests that microbial infection or injection of microbial products such as PAMPs or recombinant inflammatory cytokines in pregnancy mice could lead to adverse pregnancy complications, including preterm birth or even fetal demise (Romero et al., 2014a). Microorganisms or their ligands such as LPS, CpG, Poly (I:C) are recognized by toll-like receptors (TLRs), to induce the production of chemokines (e.g., IL-8, and C-C motif legend 2 (CCL2), cytokines (e.g., IL-1β, and TNF-α), which act on the prostaglandins and proteases to induce the common pathway of parturition (**Figure 3**, **Table 2**) (Racicot et al., 2016; Yockey et al., 2018). Indeed, murine models revealed that microbial ligands or recombinant cytokines are likely to elicit miscarriage and preterm labor (Gonzalez et al., 2011), and can be used as a predictive biomarker of the onset of preterm labor (Romero et al., 2014a), emphasizing the role of microbial induced inflammation in pregnancy complications. These studies, when taken together, highlighted the role of microbial-induced inflammation in pregnancy complications and congenital disease.

**Table 2:** Roles of cytokines in human pregnancy complications.

Cytokines	Pathological roles in pregnancy	References
IFN-α	Secreted as part of the immune response to modulate associated molecular patterns (DAMPs) or pathogen associated molecular patterns (PAMPs) Contributes to the establishment and maintenance of successful pregnancy, mediating endometrial vascular remodeling and angiogenesis at the maternal-fetal interface Overexpression correlates with viral infection or influence the placental development after ZIKV infection Overexpression toxic to early embryo development	(Chesler and Reiss, 2002; Mogensen, 2009; Murphy et al., 2009; Yockey and Iwasaki, 2018; Ni and Lu, 2018)
IFN-γ	Initiates endometrial vasculature remodeling and contributes to the normal health of the decidua Secreted in the uterus during early pregnancy. Overexpression prevents implementation and are toxic to the embryo Induce the placental damage after Malaria or Toxoplasma infection	(Murphy et al., 2009)
IL-1β	Sufficient to induce smooth muscle contraction in the uterus and preterm labor Induces abnormal lung and neurological development	(Sadowsky et al., 2006; Hogmalm et al., 2014)
IL-2	Overexpression modulates the pregnancy complication such as preeclampsia	(Hamai et al., 1997)
IL-6	Mediates embryo implantation and placental development Overexpression can mediate abnormal brain development	(Prins et al., 2012)
IL-10	Plays a pivotal role in the maternal immune tolerance for survival of an allogeneic fetus.	(Murphy et al., 2005)
IL-15	Convert decidual NK cells and macrophages to decidual phenotypes, including reduced cytotoxicity and secretion of angiogenic factors	(Ashkar et al., 2003; Chavan et al., 2016)
IL-17	Modulates the production of other pro-inflammatory cytokines Overexpression can mediate abnormal brain development	(Choi et al., 2016)
TNF-α	Key cytokine to modulate responses against infection TNF-α concentrations increase as gestation progresses albeit not excessively and may support the increased metabolic needs associated with pregnancy. Important regulator of normal cell function, influencing vital biological processes including cell proliferation, apoptosis, and the production of other cytokines such as IL-6 Overexpression can induce preterm labor and neural tube defects Overexpression also toxic to early embryo development	(Baud and Karin, 2001; Waters et al., 2013; Spence et al., 2021)

## 5 Future Directions

Although technological advances over the past decade have made significant advances on multiple fronts, including a better understanding of molecular mechanisms, more precise diagnostics, and significantly improved therapeutic outcomes, the increasing incidences of pregnancy-related complications continue to pose daunting challenges in understanding their underlying pathogenesis, host-pathogen interaction at the maternal-fetal interface. As the incidence of maternal infections and associated pregnancy complications rises, a better understanding of the developmental events that result in host-pathogen interaction at the maternal-fetal interface and aberrant immune response is critical for the development of rational intervention strategies. With the help of advanced molecular techniques, the TORCH pathogens and their ability to cross the maternal-fetal barrier to cause congenital fetus disease, which was first proposed decades ago, have now been expanded to include emerging maternal infections and the effects of microbial dysbiosis.

Despite the progress made, there are still many unanswered and widely debated questions. For example, how the placental barrier remains uncompromised to multiple microbial pathogens that cause maternal systemic illness and bacteremia, such as methicillin-resistant *Staphylococcus aureus*, *E. coli*, *SARS-CoV2* virus, while other pathogens have mastered a variety of evasion mechanisms leading to serious maternal and fetal complications? Another controversial question is the whether the placenta harbors its own microbiome or not? (Aagaard et al., 2014), and how/when does the priming of the fetal immune system with the maternal microbiome occur? (Wampach et al., 2018; de Goffau et al., 2019). The intriguing question now is, what levels of proinflammatory cytokines are required systematically or locally at the maternal-fetal interface to modulate placental integrity and allow vertical transmission of pathogens? Finally, how does maternal dysbiotic microbiota influence the maternal-fetal interface or immune response to cause pregnancy complications? While emerging multi-omics have provided us with comprehensive information about the maternal microbiome (Jehan et al., 2020; Kumar et al., 2021a), their translational impact on women’s health is still far from being achieved and requires more research.

Future research into the mechanism of host-pathogen interaction at the maternal-fetal interface, as well as how these interactions modulate immune responses and placental integrity, will have broader implications in understanding the mechanism of adverse pregnancy complications, such as miscarriage, preterm birth, and vertical transmission of pathogens. Additionally, it may lead to future therapeutic strategies to improve maternal health and prevent vertical transmission of pathogens. Advanced, cutting-edge statistical models, as well as high-throughput molecular multi-omics techniques, can be used to integrate various datasets for assessing their role in biological processes (Kumar et al., 2021b). It should be noted that numerous specific microbial therapies, such as bacteriophage or narrow-spectrum therapies that kill the specific pathogen without affecting other health microbes, are being developed and proving to be effective (Kumar et al., 2018; Brives and Pourraz, 2020). Studies are currently being conducted to determine whether these strategies will be effective for TORCH (Rodriguez-Melcon et al., 2018). Next-generation mRNA vaccines to control different maternal infections are being actively explored (Healy et al., 2019; Kumar and Al Khodor, 2021). These efforts can ultimately facilitate the design of targeted strategies to engineer the vaginal microbiota to lead to antibiotic-sparing strategies to modulate and restore a robust vaginal micro-environment, which may ultimately improve the reproductive health of women and their children.

## Author Contributions

MK, MS and SK Conceptualization, MK and SA. Writing—original draft preparation, MK and SA. Writing—review and editing, MK, MS and SA. All authors have read and agreed to the published version of the manuscript.

## Funding

This research was funded by Sidra Medicine, Qatar, grant number SDR400161, and the APC was funded by Research Department, Sidra Medicine, Qatar.

## Conflict of Interest

The authors declare that the research was conducted in the absence of any commercial or financial relationships that could be construed as a potential conflict of interest.

## Publisher’s Note

All claims expressed in this article are solely those of the authors and do not necessarily represent those of their affiliated organizations, or those of the publisher, the editors and the reviewers. Any product that may be evaluated in this article, or claim that may be made by its manufacturer, is not guaranteed or endorsed by the publisher.
